# Chronic Intermittent Mild Whole-Body Hypothermia Is Therapeutic in a Mouse Model of ALS

**DOI:** 10.3390/cells10020320

**Published:** 2021-02-04

**Authors:** Lee J. Martin, Mark V. Niedzwiecki, Margaret Wong

**Affiliations:** Departments of Pathology, Division of Neuropathology, Neuroscience, and Anesthesiology and Critical Medicine and the Pathobiology Graduate Training Program, Johns Hopkins University School of Medicine, 558 Ross Building, 720 Rutland Avenue, Baltimore, MD 21205-2196, USA; niedzmark@yahoo.com (M.V.N.); mwong14@jhmi.edu (M.W.)

**Keywords:** motor neuron, mitochondrial permeability transition pore, cyclophilin D, adenine nucleotide translocase, therapeutic hypothermia

## Abstract

Amyotrophic lateral sclerosis (ALS) is a fatal neurodegenerative disease that causes motor neuron degeneration. There are no cures or effective treatments for ALS. Therapeutic hypothermia is effectively used clinically to mitigate mortality in patients with acute acquired brain injury and in surgical settings to minimize secondary brain injury. The efficacy of therapeutic hypothermia in chronic neurodegenerative disorders has not been examined. We tested the hypothesis that mild hypothermia/cold acclimation is therapeutic in a transgenic mouse model of ALS caused by expression of mutated human *superoxide dismutase-1* gene. At presymptomatic stages of disease, body temperatures (oral and axial) of mutant male mice were persistently hyperthermic (38–38.5 °C) compared to littermate controls, but at end-stage disease mice were generally hypothermic (36–36.5 °C). Presymptomatic mutant mice (awake-freely moving) were acclimated to systemic mild hypothermia using an environmentally controlled chamber (12 h-on/12-off or 24 h-on/24 h-off) to lower body temperature (1–3 °C). Cooled ALS mice showed a significant delay in disease onset (103–112 days) compared to normothermia mice (80–90 days) and exhibited significant attenuation of functional decline in motor performance. Cooled mice examined at 80 days had reduced motor neuron loss, mitochondrial swelling, and spinal cord inflammation compared to non-cooled mice. Cooling attenuated the loss of heat-shock protein 70, mitochondrial uncoupling protein-3, and sumoylated-1 (SUMO1)-conjugated proteins in skeletal muscle and disengaged the mitochondrial permeability transition pore. Cooled ALS mice had a significant extension of lifespan (148 ± 7 days) compared to normothermic mice (135 ± 4 days). Thus, intermittent systemic mild hypothermia is therapeutic in mouse ALS with protective effects manifested within the CNS and skeletal muscle that target mitochondria.

## 1. Introduction

Amyotrophic lateral sclerosis (ALS) is a fatal neurodegenerative disease that causes skeletal muscle paralysis, respiratory failure, and death generally within 3–5 years after symptom onset [1,2]. Muscle weakness and fasciculation are early clinical signs. The cause of the morbidity is progressive skeletal muscle denervation and degeneration and loss of function and degeneration of upper motor neurons (MNs) in the cerebral cortex and lower MNs in the brainstem and spinal cord [2,3]. Aging and heterogeneous gene mutations are risk factors for ALS. Most ALS cases are sporadic with uncommon inheritance patterns. Burgeoning knowledge on the putative molecular mechanisms driving disease have not yet translated to effective treatments for ALS [2]. Only riluzole and edaravone are Food and Drug Administration (FDA) approved for the treatment of patients with ALS. Their effects are marginal and sometimes controversial [2].

The translation of basic research on brain and spinal cord injury and degenerative disorders to clinical medicine has been generally unsuccessful [2]. One exception to these disappointments is the use of targeted temperature management (therapeutic hypothermia), which is now the standard of care for neonatal hypoxic-ischemic encephalopathy and is being used in adult cardiac arrest, hypothermic circulatory arrest, and is being explored for the clinical potential in the central nervous system (CNS) trauma [4]. The mechanisms of action of therapeutic hypothermia are diverse, including protection against excitotoxicity, oxidative stress, proteinopathy, cell death, and inflammation [4]. Interestingly, these mechanisms of injury have been identified consistently in cell and animal models of ALS and in human ALS [3]. Apropos to the idea of directing targeted temperature management to ALS therapy is the evolving observation that ALS patients are hypermetabolic [5]. The exciting possibility that therapeutic hypothermia has application to chronic neurodegenerative disease has not been examined, despite the broad overlap in putative mechanisms of neuronal injury with other experimental and clinical brain injury settings where hypothermia has proven clinically translatable. We therefore tested the hypothesis that chronic, intermittent, whole-body, mild hypothermia has therapeutic efficacy in a transgenic mouse model of ALS. We found that periodic systemic mild hypothermia has therapeutic benefits in a severe mouse model of ALS with actions affecting mitochondria in the spinal cord and skeletal muscle.

## 2. Materials and Methods

### 2.1. Mice

We used transgenic (tg) mice expressing human mutant superoxide dismutase-1 (hSOD1) containing the Gly93→Ala substitution (hSOD1-G93A tg mouse). A colony existing in the lab was used. The original mouse line (line G1H, B6SJL-TgN-SOD1-G93A^1Gur^) [6] was purchased from Jackson Laboratory (Bar Harbor, ME). The hSOD1-G93A tg mouse line has been used widely by us [7,8,9,10] and others [11] as the major therapeutic testing animal model of ALS. These mice express a high copy number of human mutant alleles (20 copies) and have a rapid disease course. The disease course has presymptomatic stages (<10 weeks of age), early symptomatic stages (10–15 weeks of age) as indicated by leg tremor and paresis and nascent motor deficits, and terminal stages of disease (16–20 weeks of age) as seen by body mass wasting and paralysis. The disease progresses usually from hindleg paresis to emergent paraplegia with some function of the forelimbs remaining. At this time, ad libitum NutriGel and chow pieces are placed in the cage, and water is available at a bottom-cage level drinking spout. hSOD1-G93A tg mice have compromised locomotor activity, but they can still crawl to access food and water. Then, mice develop severe paraplegia. In this study, the end-stage disease was defined as the complete lack of locomotor activity, detected within 6 h after onset, at which time the mice were euthanized. Non-transgenic (non-tg) littermates were controls. The institutional Animal Care and Use Committee approved the animal protocols.

### 2.2. Tg Mice and Cold-Acclimation Protocol, Testing, and Triage for Endpoints

Cohorts of tg mice expressing mutated hSOD1-G93A were bred and identified by genotyping of tail DNA as described [7,8,12,13]. Mice were housed generally 4–5 mice per cage with ad libitum food and water. hSOD1-G93A tg mice were randomized into ambient normothermic (euthermic) (NT, *n* = 25) and cold-acclimated hypothermic (HT, *n* = 25) groups. The initial randomization was accomplished using the envelope method with knowledge of litter origin so that individuals from the same litter could be distributed across groups. The mice were observed twice a day through late symptomatic stages of disease and then 4–5 times a day from late symptomatic stages to end-stage disease. Body temperatures were taken with a probe (rectal, axillary, and oral). Cold-acclimation and hypothermia were induced and maintained using a cooling chamber (8–10 °C, 12 h in/12 h out) starting at 8 weeks of age and continuously thereafter. Precooling body temperatures among hSOD1-G93A mouse groups did not differ significantly (*p* > 0.05). Mouse rewarming was slow and spontaneous at room temperature. At room temperature, motor activity was tested on a voluntary activity running wheel (Harvard Apparatus, Holliston, MA, USA) as described [8,10,12,13]. Disease onset was assessed quantitatively by a running wheel activity deficit and descriptively by hindlimb paresis. For therapeutic efficacy testing, mice survived to the end-stage disease. A cadre of mice (12/group) was killed before the end-stage at 12 weeks of age to assess the efficacy of hypothermia using histological and biochemical endpoints in the spinal cord and skeletal muscle.

### 2.3. Spinal Cord and Neuromuscular Pathology

Age-matched NT and HT treated hSOD1-G93A tg mice and non-tg mice (*n* = 6 per group) at 12 weeks of age were deeply anesthetized and, after left mid-thoracotomy, without damaging the diaphragm, subjected to whole-body perfusion-fixation by cardiac puncture with a butterfly needle. Mice were exsanguinated with ice-cold 100 mM phosphate-buffered normal saline (PBS, pH 7.4) followed by fixation with 4% paraformaldehyde prepared in phosphate buffer (pH 7.4). After perfusion-fixation, mouse bodies were wrapped in aluminum foil and stored at 4 °C overnight and then the spinal cord and diaphragm were removed from each mouse. Spinal cords were cryoprotected (20% glycerol-PBS) before they were frozen-sectioned (40 μm) transversely using a sliding microtome. Serial tissue section arrays were stored individually in 96-well plates in antifreeze buffer. The diaphragm was removed as a complete tissue sheet [14] and placed in PBS at 4 °C until processed for neuromuscular junction (NMJ) visualization.

Nissl-stained transverse sections of the lumbar spinal cord were used to count the number of motor neurons (MNs) in NT and HT treated hSOD1-G93A tg mice and in age-matched littermate non-tg mice. Spinal cord sections were selected with a random start and then systematically sampled (every 10th section) to generate a subsample of sections from each mouse L3–L5 spinal cord that was mounted on glass slides and stained with cresyl violet for cell counting. Nissl-stained MNs in the ventral horn were counted by individuals blinded to the experimental treatment, using strict morphological criteria, in digital images acquired with a Nikon microscope at 200× magnification. These criteria included a round, open, pale nucleus (not condensed and darkly stained), globular Nissl staining of the cytoplasm, and a diameter of 20–40 μm. With these criteria, astrocytes, oligodendrocytes, and microglia were excluded from the counts, but these counts are likely to estimate the combined populations of α- and γ-MNs. Inter-rater reliability was assessed (*K* = 0.9).

Immunoperoxidase immunohistochemistry with diaminobenzidine as chromogen was used to assess mitochondrial pathology, specifically within spinal cord MNs, and also spinal cord inflammation. Tissue sections were selected with a random start and then systematically sampled (every remaining 10th section) to generate a subsample of sections from each mouse spinal cord. Free-floating spinal cord sections from NT and HT treated hSOD1-G93A tg mice and age-matched non-tg mice were stained with antibodies to the mitochondrial matrix protein superoxide dismutase-2 (SOD2) (Stressgen) as described [7,8,13]. MN perikaryal SOD2-positive mitochondria were counted at the base of the primary dendrites near the nucleus at 1000×. Mitochondrial diameters within MNs were measured at 1000× by an individual blinded to experimental history using ocular filar micrometry. This light microscopy quantitative assessment has been described in detail [7]. Briefly, at 1000× oil magnification the major axis of individually resolved SOD2-positive mitochondria are measured using a calibrated ocular micrometer. Microglia were stained using antibody to ionized calcium binding adaptor molecule 1 (Iba1) as described [8]. Negative control conditions for immunohistochemistry were tissue sections incubated with isotype specific non-immune immunoglobulin G (IgG) at an equivalent concentration, and for the same time as the primary antibody, and similar same time batch staining with secondary antibody, peroxidase antiperoxidase complex, and diaminobenzidine/hydrogen peroxide.

Mouse diaphragms were studied as whole mount preparations [14]. Diaphragm motor endplates were visualized with Alexa 594-conjugated α-bungarotoxin (αBTX, Invitrogen, Molecular Probes) as described [15]. MN axons were visualized in two ways. We generated double tg mice by crossing hSOD1-G93A tg mice with B6. Cg-tg Hlxb9-gfp1^Tmj/j^ mice expressing enhanced green fluorescent protein (eGFP) driven by the mouse homeobox 9 (Hb9) promoter [16]. Dual labeling was also done to visualize MN distal axons and their synaptic terminals in skeletal muscle by immunofluorescent detection of neurofilament protein using a monoclonal antibody (SMI-32, Convance, Emeryville, CA) and confocal microscopy as described [15]. The immunofluorescent labeling for neurofilament was used to determine whether the αBTX-labeled motor endplates were innervated. Confocal microscope images of the typical band distributions of motor endplates in diaphragm [14] were scored as innervated (normal) if there was overlap with the axon terminal or denervated (unoccupied) if the endplate was not associated with an axon. NMJ imaging and scoring were performed by individuals unaware of mouse treatment.

### 2.4. Immunoblotting and Immunoprecipitation (IP)

Western blot analysis was done to examine protein levels of heat shock protein-70 (HSP70), mitochondrial uncoupling protein-3 (UCP3), and protein sumoylation (SUMO1) in skeletal muscle. We selected these proteins as targets to study because they are known to be stress-responsive, and we anticipated them to be sensitive biomarkers of body thermal changes and manipulations. NT and HT hSOD1-G93A tg mice and age-match non-tg littermate control mice (*n* = 4–6/group) were deeply anesthetized with a lethal dose of chloral hydrate and decapitated for rapidly harvesting forelimb and hindlimb skeletal muscle. These samples were minced and homogenized with a Brinkmann polytron in ice-cold 20 mM Tris HCl (pH 7.4) containing 10% (*w*/*v*) sucrose, 200 mM mannitol, complete protease inhibitor cocktail (Roche), 0.1 mM phenylmethylsulfonyl fluoride, 10 mM benzamidine, 1 mM EDTA, and 5 mM EGTA. Crude homogenates were sonicated for 15 sec and then centrifuged at 1000× *g* for 10 min (4 °C). The supernatant was centrifuged at 54,000× *g* for 20 min (4 °C) to yield soluble (S2) and mitochondria-enriched pellet (P2) fractions. This subcellular fractionation protocol has been verified [17,18]. The pellet fraction was washed (twice) by trituration in homogenization buffer followed by centrifugation and then finally resuspended in homogenization buffer (without sucrose) supplemented with 20% (*w*/*v*) glycerol. Protein concentrations were measured by a Bio-Rad protein assay with bovine serum albumin as a standard.

Soluble proteins from skeletal muscle were subjected to sodium dodecyl sulfate polyacrylamide gel electrophoresis (SDS-PAGE) and transferred to nitrocellulose membrane by electroelution as described [18]. For IP prior to SDS-PAGE, skeletal muscle mitochondrial-enriched protein extract (500 µg) was input for 5 µg adenine nucleotide translocator (ANT) monoclonal antibody (clone 5F51BB5AG7, Mitoscience, Eugene, OR, USA) or cyclophilin D (CyPD) monoclonal antibody (clone E11AE12BD4, Mitoscience, Eugene, OR, USA) followed by agarose-conjugated protein A (Pierce) for capture. Negative control conditions were tissue homogenates immunoprecipitated with isotype specific non-immune IgG, with PBS but no IgG, and IP with specific primary antibody but with no homogenate input. Final total IP samples were treated with the sample buffer and boiled, and then were subjected to SDS-PAGE and Western blot. The reliability of sample loading and electroblotting in each experiment was evaluated by staining nitrocellulose membranes with Ponceau S before immunoblotting and, for IP, detection of the IgG heavy chain band. If the transfer was not uniform, blots were discarded, and gels were run again. Blots were blocked with 2.5% nonfat dry milk with 0.1% Tween 20 in 50 mM Tris-buffered saline (pH 7.4), then incubated overnight at 4 °C with antibodies to HSP70 (clone BRM-22, Sigma, St. louis, MO), UCP3 (product number U7757, St. Louis, MO), SUMO1 (R & D Systems, Minneapolis, MI, USA), ANT (Mitoscience, Eugene, OR, USA) [8], or CyPD (Mitoscience, Eugene, OR, USA) [8]. For standard Western blotting of single target proteins, the antibodies were used at concentrations for visualizing protein immunoreactivity within the linear range. As an additional protein loading control blots were reprobed with monoclonal antibody (clone 6C5, RDI) to glyceraldehyde phosphate dehydrogenase (GAPDH). After the primary antibody incubation, blots were washed and incubated with horseradish peroxidase-conjugated secondary antibody (0.2 μg/mL), developed with Pierce enhanced chemiluminescence (ThermoFisher Scientific, Rockford, IL), and imaged with a digital imager (Bio-Rad ChemiDoc System, Hercules, CA) or exposed to X-ray film.

### 2.5. Data Analysis

The mouse group sizes were based on prior calculations of power [7,8,12,13]. Observers were blinded to treatment in most instances. The values shown in the graphs represent the mean ± standard deviation. For histological data, group means and variances were evaluated statistically by a one-way ANOVA and a Student’s *t*-test. Time-to-event measures (disease onset and survival duration) were analyzed using the Kaplan–Meier survival fit analysis and log rank test. The Cox proportional hazards model was used to analyze the effect of cooling on survival and to determine the hazard ratio (HR). There was no censoring of mice due to treatment-related deaths. A one-way ANOVA followed by a Tukey post-hoc test were used for statistical comparisons for time-to-event measures.

### 2.6. Photography and Figure Construction

The original images used for histology figure construction were generated using digital photography. Digital images were captured as TiF files using a SPOT digital camera and SPOT Advanced software (Diagnostic Instruments) or a Nikon digital camera (DXM1200) and ACT-1 software. Images were altered slightly for brightness and contrast using ArcSoft PhotoStudio 2000 or Adobe Photoshop software without changing the content and actual result. Western blot figures were generated from digital primary images or scanned X-ray films. Figure composition was done using CorelDraw X5 software with final figures being converted to TiF files. Files of composite figures were adjusted for brightness and contrast in Adobe Photoshop.

## 3. Results

### 3.1. hSOD1-G93A Tg Mice Are Chronically Febrile

**Figure 1:**
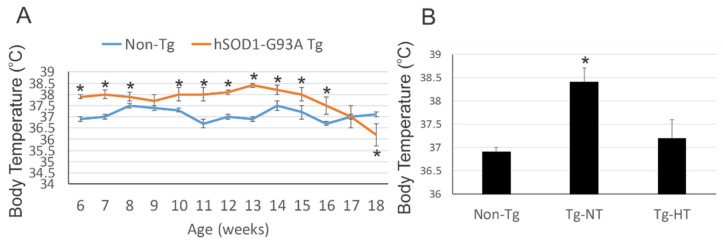
Compared to non-tg mice, male hSOD1-G93A tg mice have elevated body temperatures that can be adjusted by environmental temperature management using cold acclimation. (**A**) Male hSOD1-G93A tg mice (*n* = 25) had significantly (* *p* < 0.05) increased body temperatures throughout their disease course compared to their male age-matched non-tg littermates. (**B**) Whole-body hypothermia (HT) was induced by housing hSOD1-G93A tg mice in a cooling chamber (12 h in/12 h out, every 24 h). Non-cooled, normothermic (NT) hSOD1-G93A tg mice were maintained at ambient room temperature. At 10 weeks of age (shown here) and thereafter, longitudinal intermittent mild whole-body cooling significantly (* *p* < 0.05) reset body temperature in hSOD1-G93A tg mice (Tg-HT) by 1–2 °C compared to NT hSOD1-G93A tg mice (Tg-NT). Non-tg mice were consistently near 36.8 °C.

Body temperatures of age-matched male hSOD1-G93A tg mice and male non-tg littermates were measured beginning at 6 weeks of age through terminal disease. At 6 weeks of age, hSOD1-G93A tg mice were significantly (*p* < 0.05) higher at 38 °C compared to non-tg controls at 37 °C (Figure 1A). There was scant major fluctuation until end-stage disease for the hSOD1-G93A tg mice when they dropped significantly (*p* < 0.05) to near 36 °C (Figure 1A).

### 3.2. Environmental Temperature Management Can Induce Mild Hypothermia in hSOD1-G93A Tg Mice

At 8 weeks of age, hSOD1-G93A tg mice were randomized into treatment groups that either remained at ambient temperature to maintain normothermic body temperatures or were exposed to cold acclimatization in environmental chambers to induce whole-body hypothermia with a protocol of 12 h in/12 h out every 24 h for the duration of their survival. Longitudinal intermittent mild whole-body cooling of hSOD1-G93A tg mice (Figure 1B, Tg-HT) significantly (*p* < 0.05) reset body temperature lower by 1–2 °C compared to normothermic hSOD1-G93A tg mice (Figure 1B, Tg-NT).

### 3.3. Whole-Body Cold Acclimation Improves Neurological Outcome and Survival of hSOD1-G93A Tg Mice

**Figure 2:**
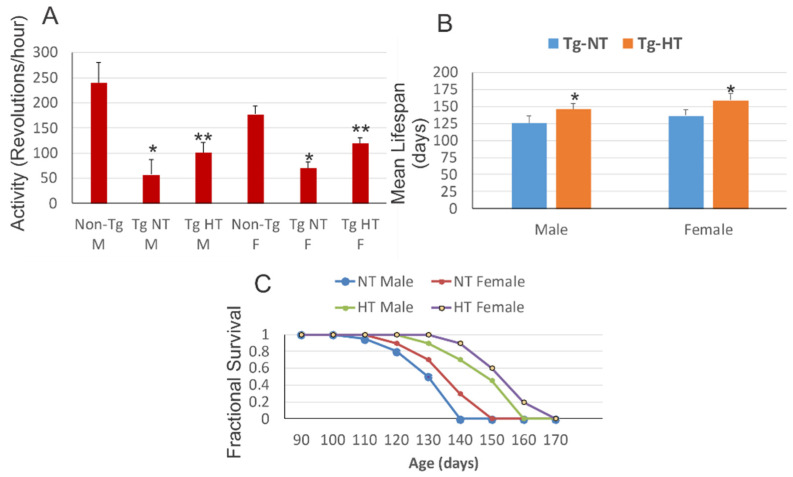
Cold acclimation mitigates neurologic decline and extends survival of male and female hSOD1-G93A tg mice. (**A**) At 10 weeks of age, male and female hSOD1-G93A tg mice that were non-cooled normothermic (Tg-NT) have a significant loss of motor activity (* *p* < 0.001) compared to sex-matched non-tg mice. Male and female hSOD1-G93A tg mice with hypothermia treatment (Tg-HT) had significantly better motor activity (** *p* < 0.05) compared to hSOD1-G93A tg mice that were non-cooled normothermic (Tg-NT). (**B**) Cooling male and female hSOD1-G93A tg mice (Tg-HT, orange) increased significantly (* *p* < 0.01) their mean lifespan compared to non-cooled normothermic hSOD1-G93A tg mice (Tg-NT, blue). (**C**) Kaplan–Meier curves showing that cooling delayed disease onset and extended overall lifespan of hSOD1-G93A tg mice. Male hSOD1-G93A tg mice with hypothermia treatment (HT Male) versus male hSOD1-G93A tg mice with no cooling (NT Male), *p* = 0.04 (HR, 95% CI, 0.71). Female hSOD1-G93A tg mice with hypothermia treatment (HT Female) versus female hSOD1-G93A tg mice with no cooling (NT Female), *p* = 0.03 (HR, 95% CI. 0.64).

At 10 weeks of age, male and female hSOD1-G93A tg mice housed at ambient temperature (Figure 2A, Tg-NT) had a significant (*p* < 0.001) reduction of motor activity compared to sex-matched non-tg littermates (Figure 2A, Non-Tg NT). Cold acclimated male hSOD1-G93A tg mice (Figure 2A, Tg-HT M) and female hSOD1-G93A tg mice (Figure 2A, Tg-HT F) showed significantly (*p* < 0.05) improved motor activity compared to hSOD1-G93A tg mice maintained at normothermic temperature (Figure 2A, Tg-NT); however, all hSOD1-G93A tg groups had significant (*p* < 0.01) motor deficits compared to age-matched non-tg mice (Figure 2A). The mean lifespans of cold acclimated male hSOD1-G93A tg mice were increased significantly (*p* < 0.01) compared to sex-matched normothermic hSOD1-G93A tg mice (Figure 2B). Overall survival of cold acclimated male hSOD1-G93A tg mice was increased significantly compared to male normothermic hSOD1-G93A tg mice (Figure 2C, log rank *p* = 0.04, HT-male versus NT-male). Cold acclimation of female hSOD1-G93A tg mice caused a slightly stronger effect on significantly increasing lifespan compared to female normothermic hSOD1-G93A tg mice (Figure 2C, log rank *p* = 0.03, HT-female versus NT-female).

### 3.4. Whole-Body Cold Cooling Protects Spinal Cord MNs and Their Mitochondria and Mitigates Inflammation in hSOD1-G93A Tg Mice

**Figure 3:**
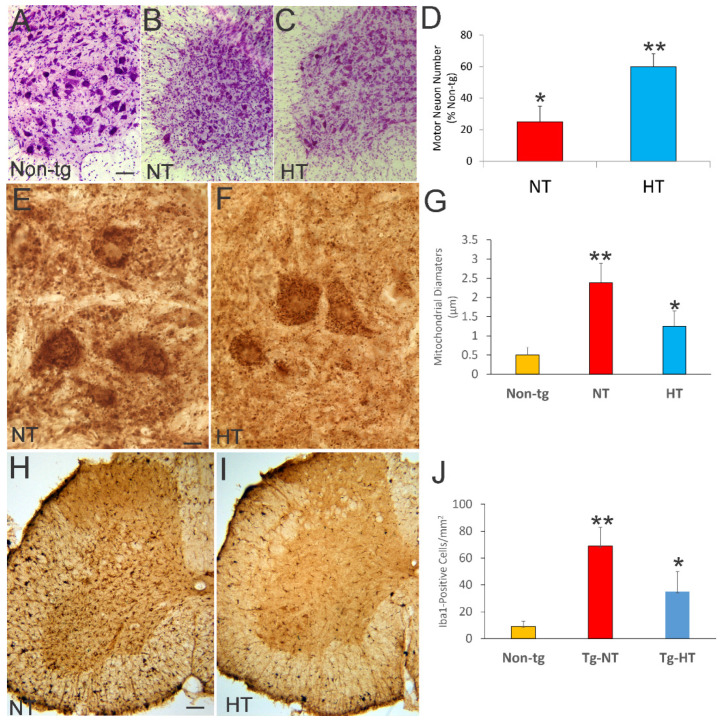
Whole-body, mild cold acclimation protected spinal cord MNs and their mitochondria and mitigated inflammation in hSOD1-G93A tg mice. Neuropathology was done on mice 12 weeks of age. (**A**–**C**) Lumbar ventral horn hemicord microphotographs of 40 µm thick spinal cord sections stained with cresyl violet (Nissl staining) showing the MN pools in a non-tg mouse (**A**) and in hSOD1-G93A tg mice that were ambient normothermic (**B**, NT) or treated with chronic intermittent mild cold acclimation hypothermia (**C**, HT). Scale bar in **A** (same for **B**,**C**) = 33.3 µm. (**D**) Counts of spinal MNs in non-tg mice and in hSOD1-G93A tg mice that were ambient normothermic (NT) or cold acclimated hypothermic (HT). Normothermic (non-cooled) hSOD1-G93A tg mice (Tg-NT) have a significant loss of MNs (* *p* < 0.001). Cooling hSOD1-G93A tg mice (Tg-HT) caused a significant (** *p* < 0.01) rescue of MNs in cold acclimated tg mice compared to non-cooled hSOD1-G93A tg mice. (**E**,**F**) Immunoreactivity (brown staining) for the mitochondrial marker SOD2 revealed the prominent mitochondriopathy in spinal cord MNs and surrounding neuropil in NT hSOD1-G93A tg mice (**E**) and the attenuation of this pathology in HT mice (**F**). Scale bar in **E** (same for **F**) = 10 µm. SOD2 shows the swelling of individual mitochondrial profiles and the disruption of mitochondria (**E**). (**G**) Graph of mitochondrial diameters (measured by ocular filar micrometry at 1000× magnification) in non-tg mice and in NT and HT hSOD1-G93A tg mice showing the significant increase in mitochondrial diameters in the NT mice (** *p* < 0.01) compared to non-tg and the significantly decreased (* *p* < 0.05) mitochondrial diameters in cold acclimated hSOD1-G93A tg mice. (**H**,**I**) Immunoreactivity (brown staining) for the microglial marker Iba1 revealed the prominent inflammation in the spinal cord in NT hSOD1-G93A tg mice (**H**) and the attenuation of this inflammation in HT mice (**I**). Scale bar in **H** (same for **I**) = 100 µm. (**J**) Counts of microglial cells in lumbar spinal cord ventral horn in hSOD1-G93A tg mice that were ambient normothermic (NT) or cold acclimated hypothermic (HT) showed significantly (** *p* < 0.01) greater numbers in NT tg mice and the significantly decreased (* *p* < 0.05) microglial cell numbers in cold acclimated hSOD1-G93A tg mice.

We evaluated cooled and non-cooled hSOD1-G93A tg mice at 12 weeks of age for neuropathology in spinal cord (Figure 3A–C). In Nissl-stained sections, non-tg mice had obvious spinal MNs with large multipolar cell bodies (Figure 3A). In non-cooled hSOD1-G93A tg mice, the MNs were depleted significantly (*p* < 0.001) 75% (Figure 3B,D) and there was secondary fulminant infiltration of small cells in the parenchyma (Figure 3B). In cold acclimated hSOD1-G93A tg mice, the MNs were more apparent compared to non-cooled mice (Figure 3B,C). Cold acclimation significantly (*p* < 0.01) rescued the number of MNs in the lumbar spinal cord of hSOD1-G93A tg mice (40% loss) compared to the 80% loss in non-cooled hSOD1-G93A tg mice (Figure 3C,D). The small cell secondary inflammation in the spinal cord parenchyma appeared attenuated in the cold acclimated mice (Figure 3C). To visualize mitochondria directly within MNs and in the neuropil at the light microscopic level we did immunostaining for SOD2 (Figure 3E,F). In non-cooled hSOD1-G93A tg mice, mitochondria were severely swollen, dysmorphic, and disrupted within MNs and in the neuropil (Figure 3E). In cooled hSOD1-G93A tg mice, mitochondria within MN cell bodies were protected from swelling (Figure 3E–G). This MN protection in hSOD1-G93A was paralleled by an attenuation of spinal cord inflammatory changes as seen by microglial Iba1 immunostaining (Figure 3H–J).

### 3.5. Whole-Body Cold Cooling Protects the NMJ

**Figure 4:**
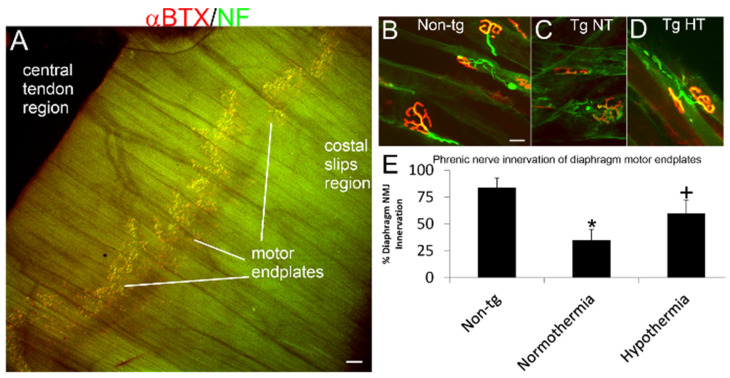
Cold acclimation preserves diaphragm NMJ integrity in hSOD1-G93A tg mice. (**A**) Low magnification image showing the band distribution of NMJs (yellow) in a mouse hemidiaphragm. Scale bar = 320 µm. (**B**–**D**) Innervation of diaphragm motor endplates (visualized by Alexa 594-conjugated α-bungarotoxin, αBTX, red) by motor neuron axons (visualized by immunofluorescence for neurofilament, NF, green) in a non-tg mouse (**B**) and in hSOD1-G93A tg mice that were ambient normothermic (**C**, Tg NT) and cold acclimated hypothermic (**D**, Tg HT) at 12 weeks of age. Scale bar (in **B**, same for **C**,**D**) = 19 µm. (**E**) Counts of innervated motor endplates in a diaphragm of non-tg mice and hSOD1-G93A tg mice that were ambient normothermic or cold acclimated hypothermic showed significant loss (* *p* < 0.01) of innervation in normothermia hSOD1-G93A tg mice and rescue (+ *p* < 0.05) of innervation in cold acclimated hypothermia hSOD1-G93A tg mice.

To assay for whether hypothermia protects NMJs in hSOD1-G93A tg mice, a whole-mount diaphragm preparation was used (Figure 4A). In non-tg mice, motor endplate innervation of diaphragm was near 100% (Figure 4B,E), while in non-cooled hSOD1-G93A tg mice endplate innervation was reduced significantly (*p* < 0.01) to only about 40% (Figure 4C,E) by 12 weeks of age. In contrast, in cold acclimated hSOD1-G93A tg mice, NMJ innervation was restored to about 65% of normal but was still significantly reduced (*p* < 0.05) compared to non-tg mouse diaphragm innervation (Figure 4D,E).

### 3.6. Whole-Body Cold Acclimation Induces Cytoprotection Mechanisms and Disengages the Mitochondrial Permeability Transition Pore (mPTP) in Skeletal Muscle of hSOD1-G93A Tg Mice

**Figure 5:**
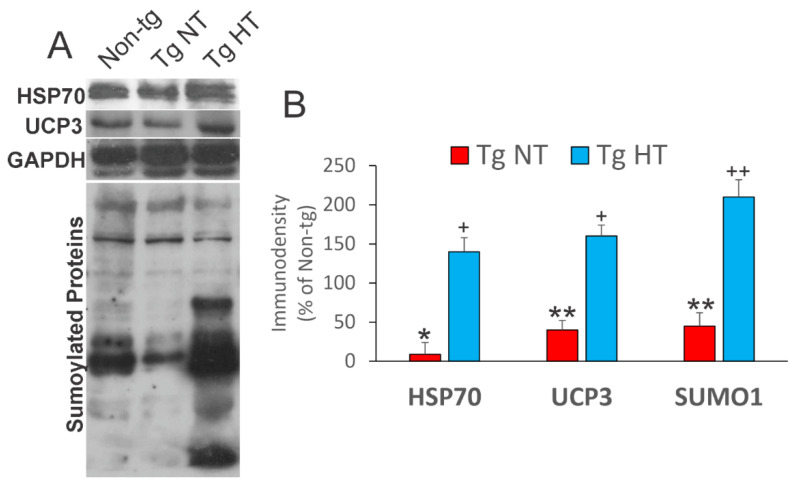
Whole-body cooling induced changes in cytoprotective proteins in the skeletal muscle of hSOD1-G93A tg mice at 12 weeks of age. (**A**) Representative Western blots for heat shock protein 70 (HSP70), uncoupling protein 3 (UCP3), and protein sumoylation (SUMO1) in hindleg muscle of non-tg mice (non-tg) and hSOD1-G93A tg mice that were cooled (Tg HT) or not cooled (Tg NT). GAPDH was used as a protein loading control. (**B**) Graph of quantitative analysis of target protein immunodensities. In NT hSOD1-G93A tg mice, HSP70 (* *p* < 0.001), UCP3 (** *p* < 0.01), and SUMO1 conjugated proteins (** *p* < 0.01) were significantly reduced compared to non-tg mice. In HT hSOD1-G93A tg mice, the levels of immunoreactivity for HSP70, UCP3, and SUMO-conjugated proteins were restored to non-tg levels or significantly exceeded non-tg levels (+ *p* < 0.05, ++ *p* < 0.01).

Skeletal muscle was interrogated for adaptive changes induced by therapeutic hypothermia. We queried heat-shock, UCP, and protein sumoylation networks. Non-cooled normothermic hSOD1-G93A tg mice (Tg-NT) had significant losses of HSP70 (*p* < 0.001), UCP3 (*p* < 0.01), and sumoylated protein (*p* < 0.01) in skeletal muscle compared to non-tg mice (Figure 5A,B). Cooled hSOD1-G93A tg mice had significant rescue and upregulation of HSP70 (*p* < 0.05), UCP3 (*p* < 0.05), and SUMO1 modified proteins (*p* < 0.01) compared to non-cooled tg mice (Figure 5A,B).

**Figure 6:**
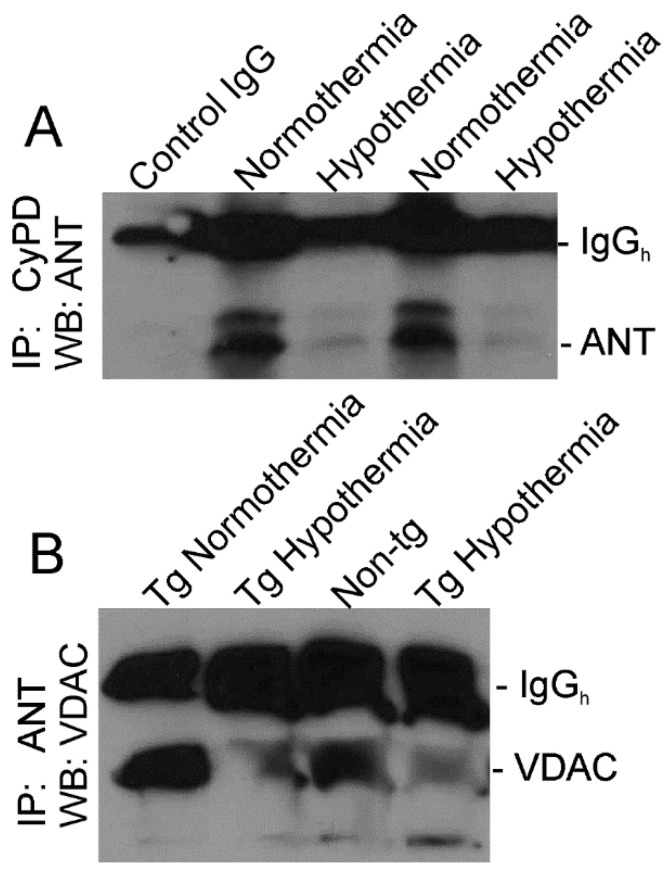
Immunoprecipitation (IP) shows diminished interaction of mPTP related proteins in skeletal muscle of hSOD1-G93A tg mice with cold acclimation at 12 weeks of age. (**A**) IP of CyPD in skeletal muscle mitochondrial-enriched fractions of hSOD1-G93A tg mice with chronic intermittent cooling (hypothermia) and without cooling (normothermia) followed by Western blotting for ANT. IP was done using monoclonal antibody to CyPD (E11Ae12BD4). The negative control was IP with an equal amount of non-immune mouse IgG1κ. (**B**) IP of ANT in skeletal muscle mitochondrial-enriched fractions of non-tg mice and hSOD1-G93A tg mice with cooling (Tg hypothermia) and without cooling (Tg normothermia) followed by Western blotting for VDAC. IP was done using monoclonal antibody to ANT (5F51BB5AG7).

We have shown previously that whole-body, non-conditional *CyPD* gene-null inactivation of the mPTP protects hSOD1-G93A tg mice from ALS [8] and effectively blocks different forms of cell death in brain and spinal cord, including neuronal apoptosis and necrosis [16]. We tested the hypothesis using IP that cooling affects the mPTP in skeletal muscle (Figure 6). CyPD, encoded by the *ppif* gene, is a peptidyl prolyl isomerase known to interact with the ANT [19]. IP for CyPD followed by Western blotting for ANT showed that cold acclimated hSOD1-G93A tg mice had attenuated CyPD–ANT interaction compared to normothermic hSOD1-G93A tg mice (Figure 6A). In addition, ANT has been shown previously to interact with VDAC to form a putative mPTP or a regulator of the mPTP [20]. IP for ANT and Western blotting for VDAC revealed that interaction of ANT and VDAC was attenuated in cold acclimated hSOD1-G93A tg mice compared to normothermic hSOD1-G93A tg mice (Figure 6B).

## 4. Discussion

We found that long-term, intermittent mild whole-body cold acclimation-hypothermia improved neurological and neuropathological outcomes and extended lifespan in a sex-related manner in a tg mouse model of ALS. Cold acclimation was more effective in females than in males for extending lifespan. Diaphragm innervation of motor endplates was improved by hypothermia. Some mechanisms related to these protective effects in vivo could be mediated by hypothermia-induced upregulation of multiple cytoprotective molecular pathways involving protein chaperones, protein posttranslational modification, and mitochondrial proteins in skeletal muscle. Importantly, modulation of proteostasis, inactivation of the mPTP, and mitigation of mitochondrial swelling could be part of the mechanisms of the beneficial effects of therapeutic hypothermia. The concept of longitudinal whole-body temperature management by cooling and thermoregulatory modifiability by ambient-environmental control tools or potential pharmaceuticals is a novel therapeutic consideration for the protection of body mitochondria and the treatment of chronic fatal neurodegenerative disease such as ALS.

We found that whole-body hypothermia acclimation delayed the onset of disease and the decline in neurological function, and the time to fatal disease in ALS mice. These effects are different from the effects of therapeutic hypothermia used for neonatal hypoxic-ischemic encephalopathy where this clinical intervention primarily reduces acute mortality [21], but many significant severe forms of morbidity persist [22,23]. In our ALS mice, mortality ultimately prevailed. We also found that cooling was more effective in female mice than in male mice with chronic disease, but clinically in newborns there are no sex differences seen with cooling [23]. Thus, the therapeutic effects of cooling appear to be temporary in this experimental setting of severe chronic neurodegenerative disease, but this delay in fatality could provide additional opportunities for adjuvant treatments to further extend lifespan in meaningful ways. For example, the treatment with riluzole and edaravone could be combined with cooling to determine if additive effects on lifespan exist. We have found additive effects with cooling and adjuvant drug treatment before in other experimental animal settings of brain injury [24]. Cold-acclimated ALS mice could also be used to screen novel adjuvant drug candidates.

This hSOD1-G93A tg mouse model of ALS is severe and is characterized by a clinical inflammatory state during the progression of disease. Evidence for inflammation is found in organs [9,25] and plasma [26]. Many of the circulating cytokines are pyrogenic [27]. This inflammatory cytokine profile is consistent with our finding that ALS mice are febrile during the disease course. Key circulating pyrogenic proinflammatory cytokines in ALS mice are TNFα and IL6 [26,28]. TNFα and IL6 can drive skeletal muscle wasting in a variety of clinical settings [29,30]. Skeletal muscle wasting is a prominent feature of human ALS and some mouse models of ALS [6,13]. Primary sentinel molecular and cellular events in skeletal muscle might be causal pathophysiology in ALS [12,13,31]. We have found during the course of disease in hSOD1 ALS mouse skeletal muscle that constitutive and inducible forms of nitric oxide (NO) synthase protein isoforms are upregulated, NO production is increased, and nitration of proteins is elevated, including key proteins at the NMJ [13]. Thus, understanding how cold acclimation affects inflammation systemically and NO pathobiology in ALS could be meaningful.

Thermoregulatory function in ALS might be aberrant [32]. ALS patients are reported to be hypermetabolic as determined by dual-energy X-ray absorptiometry [5]. A previous study has reported that a human mutant SOD1 mouse, different from the one used here, cannot maintain body temperature with an acute cold challenge [33]. Human mutant SOD1 mice also show significant attrition of brown fat during disease [13]. This information is consistent with our finding on the modifiability of ALS mouse body temperature with cold acclimation. As part of the clinical phenotype ALS, the autonomic nervous system seems to be abnormal in patients [34]. Moreover, small fiber peripheral neuropathy has been described in ALS [35,36]. The sympathetic nervous system innervates brown fat reservoirs [32]. The concept that thermoregulatory, metabolic, autonomic, and peripheral sensory pathways are perturbed in ALS is consistent with the finding that the anterior hypothalamus is atrophic in human ALS in relation to the body mass index [37]. All of these changes signal that the pathology and disease mechanisms are much more complex than cell autonomous motor neuron disease in ALS. Chronic intermittent mild whole-body hypothermia could be modifying a variety of these mechanisms [3], including hypermetabolism, mitochondriopathy, and inflammation, within the CNS, peripheral nervous system, and skeletal musculature.

We discovered that mild hypothermic cold acclimation induced cytoprotective mechanisms in the skeletal muscle of ALS mice. Cold acclimation modulated molecular chaperone, posttranslational, and mitochondrial networks. We found that HSP70 was depleted in ALS mouse skeletal muscle at a mid-symptomatic stage of disease (12 weeks of age). HSP70 is the main cytosolic effector of the heat shock response [38]. Cooling ALS mice restored levels of HSP70 in skeletal muscle. This restoration of HSP70 could be related to the survival effects of cooling on ALS mice because exogenous delivery of HSP70 to skeletal muscle extends lifespan in the same mouse model of ALS [39]. Regarding protein posttranslational modification, ALS mouse skeletal muscle also showed a global loss of SUMO1-conjugated proteins at 12 weeks of age. Our finding of cooling-induced accumulation of SUMO1-conjugated proteins in skeletal muscle is consistent with other work showing that moderate hypothermia (30 °C) increases protein sumoylation in adult rat brain [40]. Our result with the rescue of SUMO1-conjugated proteins in the skeletal muscle of cooled ALS mice is intriguing because sumoylation of DNA damage repair protein networks has been shown to facilitate DNA repair in cell culture [41]. Others and we [42,43,44,45,46] have proposed that DNA damage could be a sentinel mechanism of pathogenesis in ALS, particularly in skeletal muscle [13]; thus, the therapeutic efficacy of cooling in ALS mice could be related to enhanced repair of DNA damage in skeletal muscle. Enforced DNA repair is directly neuroprotective, thus demonstrating that DNA damage accumulation is a driver of neuronal cell death in target deprivation settings, perhaps occurring in ALS [13,47], rather than a consequence of dying neurons [48]. In clinical settings, cooling reduces DNA damage in infants with hypoxic ischemic encephalopathy [49]. We also found a downregulation of UCP3 in the skeletal muscle of SOD1 mice maintained at ambient temperature at 12 weeks of age, and a bolstering of UCP3 levels in SOD1 mice with intermittent cold acclimation. The downregulation UCP3 protein level in non-cooled mice is consistent with a compensatory response to a systemic febrile state [50]. This loss of UCP3 could also be related to disease mechanisms because it can lead to increased production of mitochondrial ROS in skeletal muscle [51]. An upregulation of UCP3 in skeletal muscle with cooling could be an adaptive attempt to counteract the cold environment through thermoregulatory and fat oxidation mechanisms [52,53]. In these ALS mice, there is significant NMJ denervation at 12 weeks of age. We are currently uncertain about the role that denervation and cooling-mediated rescue of NMJ integrity might play in modulating the levels of HSP70, UCP3, and SUMO1-conjugated proteins.

We found that cold acclimation affects the mPTP in skeletal muscle and mitochondrial swelling in MNs of ALS mice. Our IP data suggests that cooling disengages molecular components of the mPTP. A key mechanism of this disinhibition could center on CyPD, which is, despite changing ideas on the core components of the mPTP, persistently described as an important regulator of the mPTP [20,54]. We found that cooling diminished the interaction of CyPD with the ANT. This finding is very interesting because homozygous deletion of the *CyPD* gene has been shown to extend the survival of two different mutant SOD1 transgenic mouse models of ALS [8]. Moreover, a small molecule inhibitor of the mPTP extends the lifespan of ALS mice [55]. Though a very different experimental design, a study using an acute model of cardiac arrest has shown that post-arrest hypothermia appears to inhibit the mPTP through a CyPD mechanism [56]. The current study reinforces the concept that mitochondriopathy and the mPTP are drivers of pathobiology in ALS [3,8,55] and that disengagement of the mPTP is a therapeutic target. Mitochondrial preservation and mPTP disengagement might be mechanisms through which therapeutic hypothermia mediates its neuroprotective effects in other settings of CNS injury such as neonatal hypoxic-ischemic encephalopathy.

## 5. Conclusions

This work took the ALS ice bucket challenge to a very different conceptual level by showing that cooling ALS mice is therapeutic. hSOD1-G93A tg ALS mice were found to be febrile, but chronic, intermittent, whole-body hypothermia was found to reset body temperature. Though precooling body temperatures were known among hSOD1-G93A tg mouse groups, they were similar prior to the assignment into normothermia and hypothermic groups. Cold acclimation appears to be acting therapeutically in part by driving adaptive mechanisms in skeletal muscle, but hypothermia could be acting at CNS, peripheral nervous system, skeletal muscle, and body fat levels [13]. A skeletal muscle action would be consistent with emerging evidence that the skeletal muscle is directly driving some disease mechanisms in ALS as has been demonstrated in other work [12,13,31]. The clinical-translational application and efficacy testing of this concept in human ALS would be non-invasive and implementable, possibly at rehabilitation spas in private and hospital settings. Cold water immersion or cryotherapy is commonplace for athletes. The cold acclimation protocols would need to be empirically determined and refined, and biomarkers of therapeutic efficacy need to be identified for human ALS. Starting point biomarkers could be based on skeletal muscle biopsy and assay for HSP70, protein sumoylation, and mPTP activation thresholds.

## Data Availability

Data and materials supporting the conclusions of this work are included herein. L.J.M. is to be contacted to request availability of these materials.
